# RACIAL SIMILARITIES AND DIFFERENCES IN FUTURE CARE PLANNING

**DOI:** 10.1093/geroni/igz038.1633

**Published:** 2019-11-08

**Authors:** Natasha L Peterson, Jeong Eun Lee

**Affiliations:** Iowa State University, Ames, Iowa, United States

## Abstract

Recent population trends for the United States show substantial increases in racial diversity as well as an increase in the older adult demographic. Accordingly, the expansion in minority older adults suggests the importance of recognizing how race affects future care planning (FCP) in late life. The aim of this paper is to investigate the racial similarities and differences in planning behavior and intentions related to end-of-life care planning. This study utilized data from the Future Care Planning intervention based on the initial assessment with 237 older adults in rural Iowa (Mage= 75.93, SD=11.14) with 73 non-White participants. Results indicated that both Whites and non-Whites had similar levels of intentions in terms of future care. However, non-Whites were less likely than Whites to engage in multiple areas of FCP including planning behavior and preparing for end-of-life (F=23.957, p<.000). These findings suggest a need for more targeted FCP assessments and interventions across race groups in rural contexts.

